# Two classes of ovarian primordial follicles exhibit distinct developmental dynamics and physiological functions

**DOI:** 10.1093/hmg/ddt486

**Published:** 2013-10-01

**Authors:** Wenjing Zheng, Hua Zhang, Nagaraju Gorre, Sanjiv Risal, Yan Shen, Kui Liu

**Affiliations:** Department of Chemistry and Molecular Biology, University of Gothenburg, Gothenburg SE-405 30, Sweden

## Abstract

In the mammalian ovary, progressive activation of primordial follicles serves as the source of fertilizable ova, and disorders in the development of primordial follicles lead to various ovarian diseases. However, very little is known about the developmental dynamics of primordial follicles under physiological conditions, and the fates of distinct populations of primordial follicles also remain unclear. In this study, by generating the *Foxl2-CreER^T2^* and *Sohlh1-CreER^T2^* inducible mouse models, we have specifically labeled and traced the *in vivo* development of two classes of primordial follicles, the first wave of simultaneously activated follicles after birth and the primordial follicles that are gradually activated in adulthood. Our results show that the first wave of follicles exists in the ovaries for ∼3 months and contributes to the onset of puberty and to early fertility. The primordial follicles at the ovarian cortex gradually replace the first wave of follicles and dominate the ovary after 3 months of age, providing fertility until the end of reproductive life. Moreover, by tracing the time periods needed for primordial follicles to reach various advanced stages *in vivo*, we were able to determine the exact developmental dynamics of the two classes of primordial follicles. We have now revealed the lifelong developmental dynamics of ovarian primordial follicles under physiological conditions and have clearly shown that two classes of primordial follicles follow distinct, age-dependent developmental paths and play different roles in the mammalian reproductive lifespan.

## INTRODUCTION

In mammals, the pool of primordial follicles serves as the source of developing follicles and fertilizable ova for the entire reproductive lifespan of the organism (1,2). Whereas the majority of primordial follicles remain in a dormant state, a limited number of primordial follicles are recruited from the resting follicle reservoir into the growing follicle pool. In recent years, the molecular mechanisms controlling the activation of primordial follicles have begun to be revealed mostly by using genetically modified mouse models, which represent extreme situations in which a particular gene is deleted (for reviews see references 3 and 4). Very little is known, however, about the development of primordial follicles under physiological conditions throughout the entire female reproductive lifespan.

Previous studies have shown that the supporting cells recruited in fetal mouse ovaries form the initial primordial follicles in the medullary region of the ovary (5,6). Once formed, these primordial follicles in the medulla are synchronously activated and become the first wave of activated follicles after birth, and they have long been considered not to contribute to fertility (5,7). The supporting cells recruited in postnatal mouse ovaries form primordial follicles in the cortical region (5,6). The cortical primordial follicles are believed to be activated gradually as a means of providing mature ova over the entire course of the animal's reproductive life (8,9).

In the current study, we have generated two tamoxifen-inducible knock-in mouse models, the *Forkhead box L2* (*Foxl2*)*-CreER^T2^* mice and the *Spermatogenesis and oogenesis-specific basic helix-loop-helix 1 (Sohlh1)-CreER^T2^* mice. These mouse models have allowed us to specifically label the first wave of activated follicles in the ovarian medulla and the primordial follicles in the cortex, respectively. We have traced the *in vivo* development of the two classes of follicles over the lifetimes of the mice, and our results show that the first wave of medullary follicles and the cortical primordial follicles are distinct populations in terms of their developmental dynamics, their contributions to the onset of puberty and their contributions to fertility during young adulthood and later in reproductive life.

## RESULTS

### Generation of a *Foxl2-CreER^T2^* knock-in mouse model for labeling the first wave of activated follicles in postnatal ovaries

Foxl2 is a forkhead transcription factor that is expressed in somatic cells of the early XX gonad and in pregranulosa and granulosa cells (10,11). To selectively label the first wave of activated follicles that appear in the ovarian medulla after birth, we generated a tamoxifen-inducible knock-in mouse model by placing an *IRES-CreER^T2^* cassette after the endogenous *Foxl2* exon to produce *Foxl2-CreER^T2^* mice (Supplementary Material, Fig. S1A and B). This mouse model allowed us to use tamoxifen to induce the labeling of granulosa cells of only the first wave of activated follicles after birth, but to avoid labeling the entire pool of follicles, as described later.

**Figure 1.**
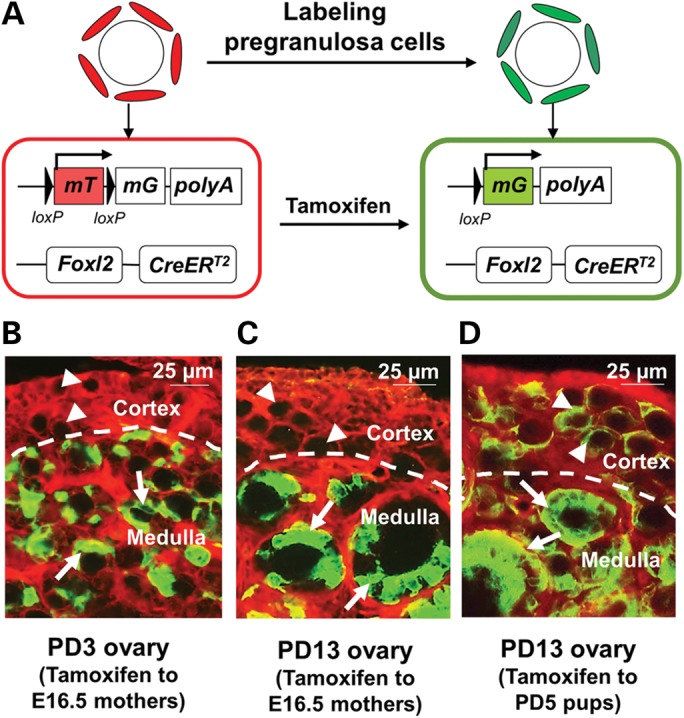
Labeling the first wave of activated follicles in *Foxl2-CreER^T2^;mT/mG* mouse ovaries. (**A**) Illustration of the tamoxifen-induced labeling of pregranulosa and granulosa cells of the first wave of activated follicles in postnatal *Foxl2-CreER^T2^;mT/mG* mice. In *Foxl2*-expressing pregranulosa and granulosa cells, the CreER^T2^ recombinase is not active and the cells express membrane-targeted Tomato (mT), a red fluorescent protein. Upon tamoxifen induction, the activated CreER^T2^ recombinase mediates the deletion of the *mT* region and switches on the expression of membrane-targeted green fluorescent protein (mG) resulting in follicles that are labeled with green pregranulosa and granulosa cells. (**B** and **C**) Efficient labeling of the first wave of activated follicles by administration of tamoxifen to pregnant females. Pregnant females were given a single intraperitoneal injection of tamoxifen at E16.5, and the ovaries of their pups at PD3 (B) and PD13 (C) were analyzed. Note that the first wave of activated follicles in the ovarian medulla was efficiently labeled (arrows) whereas primordial follicles in the ovarian cortex were not labeled (arrowheads). Although not all pregranulosa and granulosa cells were labeled by the low dosage of tamoxifen used, a follicle was considered successfully labeled as long as one somatic cell turned green. (**D**) Labeling of all follicles by tamoxifen injection at PD5. The female mice were given a single intraperitoneal injection of tamoxifen at PD5 and were analyzed at PD13. This protocol of tamoxifen administration led to the labeling of all follicles, including the first wave of follicles (arrows) and the cortical primordial follicles (arrowheads).

We crossed the *Foxl2-CreER^T2^* mice with the *mT/mG* reporter mice. As illustrated in Figure 1A, labeled pregranulosa cells show a switch from red fluorescence (mT) to green fluorescence (mG) (12). **Throughout the manuscript, we refer to green cells as ‘labeled’ and red cells as ‘unlabeled’.** As shown in Figure 1B, when we gave a single injection of tamoxifen to pregnant females at Embryonic Day (E) 16.5 [15 mg/kg body weight (BW), ∼600 μg per mouse] all activated follicles in Postnatal Day (PD) 3 ovaries of the offspring showed labeling of the granulosa cells with green fluorescence (Fig. 1B, arrows). The primordial follicles in the cortical region were unlabeled and their pregranulosa cells still expressed red fluorescence (Fig. 1B, arrowheads). Similarly, at PD13, almost all growing follicles in the ovarian medulla were labeled with green fluorescent granulosa cells (Fig. 1C, arrows), whereas the primordial follicles in the cortical region remained unlabeled and showed red fluorescence (Fig. 1C, arrowheads).

As a comparison, when *Foxl2-CreER^T2^;mT/mG* pups were injected with tamoxifen at PD5 (15 mg/kg BW, ∼60 μg per pup), which is the time when the cortical primordial follicles are being formed (6), we could label both the granulosa cells of all growing follicles (Fig. 1D, arrows) and the pregranulosa cells of all cortical primordial follicles (Fig. 1D, arrowheads) at PD13. As negative controls, no labeling of cells was seen in *Foxl2-CreER^T2^;mT/mG* mice injected with vehicle alone (Supplementary Material, Fig. S2A) or in *mT/mG* mice injected with tamoxifen (Supplementary Material, Fig. S2B). In addition, the development of ovarian follicles was not influenced by embryonic exposure to tamoxifen (see Supplementary Material, Text and Supplementary Material, Fig. S3A–C).

These results clearly indicate that by giving tamoxifen to pregnant females at E16.5, we have specifically labeled the first wave of activated follicles in the ovarian medulla, but have avoided labeling the primordial follicles in the ovarian cortex.

### The first wave of activated follicles contribute to the onset of puberty and to fertility in young adulthood

The specific labeling of the first wave of activated follicles allowed us to follow their *in vivo* development to different ages and to determine the time periods that they remain in the mouse ovaries as well as their contributions to the onset of puberty and to fertility.

#### Tracing the first wave of activated follicles to the onset of puberty (PD23)

**Figure 2.**
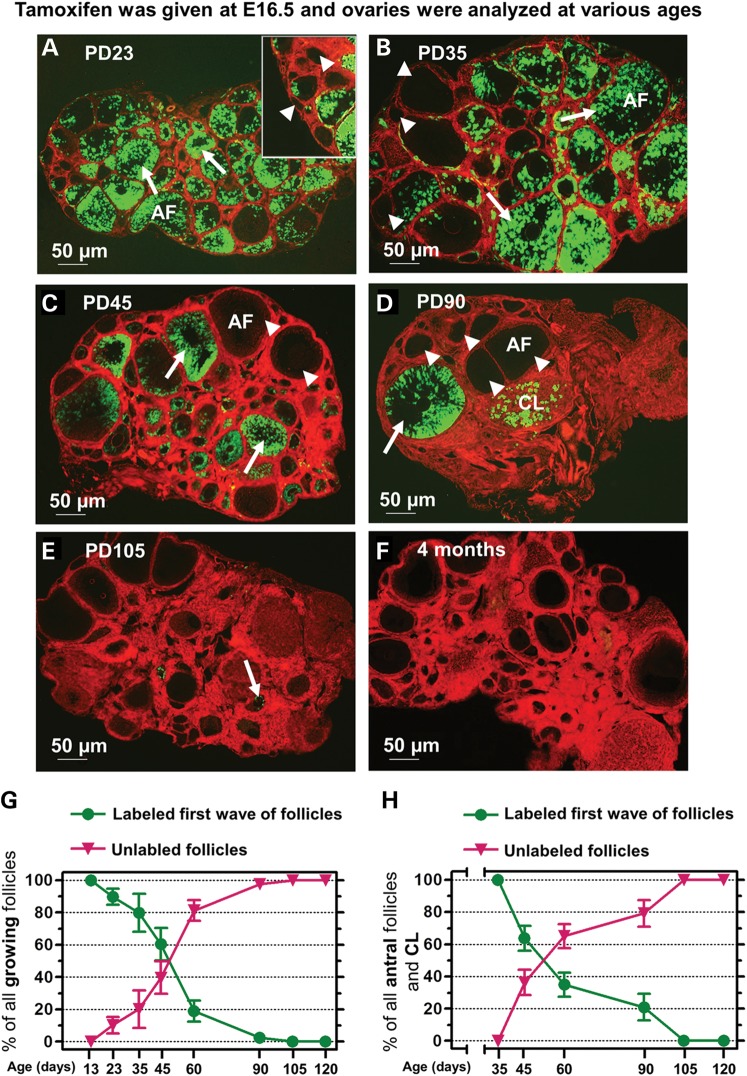
Tracing the development of the first wave of activated follicles in *Foxl2-CreER^T2^;mT/mG* mouse ovaries. Tamoxifen was administered to pregnant females at E16.5. The *Foxl2-CreER^T2^;mT/mG* pups born to these females were sacrificed at different ages to analyze their ovaries. (**A** and **B**) Most of the growing follicles in ovaries collected from PD23 (A) and PD35 (B) mice show labeled green granulosa cells (arrows), indicating that they have developed from the first wave of activated follicles. The presence of labeled antral follicles (AF) indicates that follicles from the first wave contribute to the onset of puberty and to fertility during young adulthood. A few unlabeled red growing follicles (arrowheads) can also be seen, indicating that they developed from the cortical primordial follicles. (**C**) At PD45, both labeled green (arrows) and unlabeled red (arrowheads) growing follicles are observed, and some unlabeled follicles have reached the antral stage (AF). (**D**) At PD90, the majority of the growing follicles, including antral follicles (AF), are unlabeled (arrowheads). The number of labeled follicles (arrow) has decreased, and most of these are at advanced stages or have differentiated into corpora lutea (CL). (**E**) At PD105, only green debris can be observed in the ovary (arrow). (**F**) At 4 months, no green fluorescent cells can be observed. (**G**) Quantification of labeled and unlabeled growing follicles at different ages. At PD105, the first wave of activated follicles becomes depleted, and the follicles grown from cortical primordial follicles become the only source of the growing follicle pool. (**H**) Quantification of labeled and unlabeled antral follicles and CL as a marker for their contributions to fertility. At PD105, no antral follicles or CL are from the first wave of follicles. In G and H, *n* = 4 for PD13, *n* = 4 for PD23, *n* = 5 for PD35, *n* = 5 for PD45, *n* = 5 for PD60, *n* = 5 for PD90, *n* = 4 for PD105 and *n* = 4 for 4 months. Values are means ± SD.

At PD23, the time when puberty begins, most of the growing follicles (including primary and further-developed follicles) contained granulosa cells with green fluorescence (Fig. 2A, arrows). This green fluorescence showed that these follicles had developed from the labeled first wave of follicles that were activated after birth (Fig. 1B and C, arrows). Some of the first wave follicles had even reached the antral stage by PD23 (Fig. 2A, AF).

Some cortical primordial follicles also started to become activated at this age. As shown in Figure 2A (inset, arrowheads), primary and secondary follicles showing red fluorescence were seen in the vicinity of the cortical region indicating that these follicles developed from the unlabeled cortical primordial follicles (Fig. 1 B and C, arrowheads).

As quantified in Figure 2G, at PD23, the first wave of follicles accounted for 89.8 ± 5.1% (mean ± SD) of the growing follicle pool. Accordingly, 10.2 ± 5.1% of the growing follicles exhibited red fluorescence showing that the cortical primordial follicles had started to replace the first wave of follicles at PD23.

Based on these results, we conclude that the majority of the growing follicles at the time of puberty onset originate from the first wave of activated follicles and are the main follicular population that contributes to the onset of puberty.

#### Tracing the first wave of activated follicles to the time of sexual maturity (PD35)

At the time of sexual maturity (around PD35), 79.8 ± 11.7% (Fig. 2G) of the growing follicles were still labeled with green granulosa cells (Fig. 2B, arrows), indicating that the first wave of follicles was still dominant in the ovary. As shown in Figure 2B (AF) and quantified in Figure 2H, 100% of the antral-stage follicles in the ovary were labeled with green fluorescence and thus had developed from the first wave of follicles. Therefore, the first wave of follicles contributed to early fertility at the time of sexual maturity.

Meanwhile, the proportion of unlabeled red growing follicles (Fig. 2B, arrowheads) had increased to 20.2 ± 11.7% (Fig. 2G), indicating that the activated cortical primordial follicles were gradually replacing the first wave of follicles.

#### Tracing the first wave of activated follicles to young adulthood (PD45–90)

At PD45, 60.4 ± 10.0% (Fig. 2G) of the growing follicles had originated from the first wave of follicles (Fig. 2C, arrows), and 39.6 ± 10.0% (Fig. 2G) of the growing follicles had originated from cortical primordial follicles (Fig. 2C, arrowheads). At this point in development, some unlabeled red follicles had developed as far as the antral stage (Fig. 2C, AF), and these made up 36.3 ± 7.9% of all antral follicles (Fig. 2H).

At PD90, the percentage of labeled green follicles (Fig. 2D, arrow) dropped to 2.4 ± 0.7% (Fig. 2G). In contrast, the unlabeled red growing follicles (Fig. 2D, arrowheads) represented 97.6 ± 0.7% of all growing follicles and had become dominant in the ovary (Fig. 2G). As quantified in Figure 2H, 20.9 ± 8.2% of all antral follicles and corpora lutea (CL) were labeled with green fluorescent cells at PD90 (Fig. 2D, arrow and CL), whereas 79.1 ± 8.2% of all antral follicles and CL contained only red fluorescent cells at this age.

These results showed that the first wave of follicles was still actively contributing to fertility in young adulthood, but follicles that developed from the cortical primordial follicles were gradually but steadily becoming the major follicular resource between PD45 and PD90.

#### Tracing the first wave of activated follicles up to 4 months of age

Only a small amount of green cellular debris was observed in the ovaries of PD105 mice (Fig. 2E, arrow). At 4 months of age, all follicles and CL were composed of unlabeled red somatic cells (Fig. 2F). Therefore, the first wave of follicles was present in the mouse ovary up to ∼3 months after birth. After that, the cortical primordial follicles served as the only source of growing follicles (Fig. 2G).

To summarize, the first wave of follicles dominated the growing follicle pool until young adulthood (PD45) and facilitated the onset of puberty and the entry into sexual maturity. Then, the proportion of the first wave of follicles had dropped to ∼20% of all growing follicles at PD60 and to ∼2% at PD90 (Fig. 2G). These results suggest that the first wave of follicles provides fertility up to 3 months of age (Fig. 2H). Notably, although the activation of the first wave of follicles was synchronous, their further development was asynchronous. Some of them grew more rapidly and reached the antral stage at PD23 (Fig. 2A, AF) whereas others developed more slowly and ovulated around PD90 (Fig. 2D, CL).

### Generation of a *Sohlh1-CreER^T2^* knock-in mouse model for labeling primordial follicles in adult mouse ovaries

**Figure 3.**
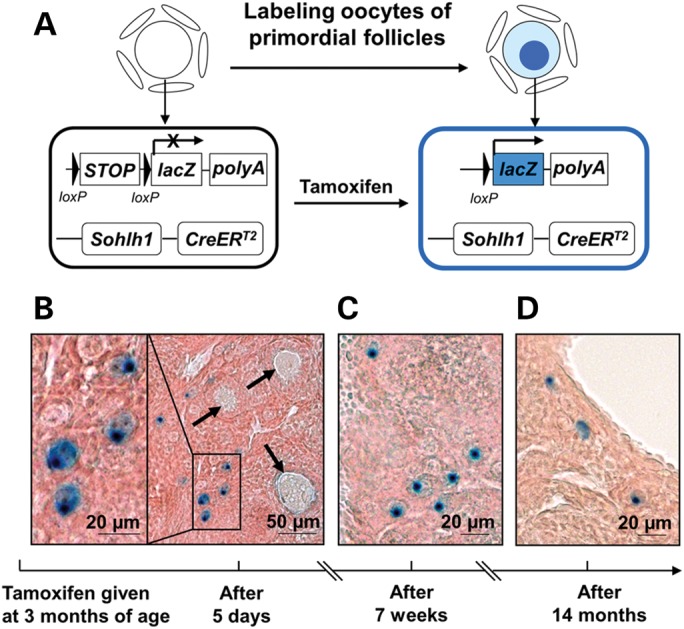
Labeling of primordial follicles in adult mouse ovaries using the *Sohlh1-CreER^T2^;R26R* mouse model. (**A**) Illustration of the tamoxifen-induced labeling of adult primordial follicles in the *Sohlh1-CreER^T2^;R26R* mice. In the oocytes of primordial follicles, the CreER^T2^ mediated by the *Sohlh1* promoter is not active and no β-galactosidase is expressed before tamoxifen is given. Upon tamoxifen administration, the CreER^T2^ recombinase becomes activated and mediates the removal of the *STOP* sequence that is in front of the *lacZ* (the gene encoding β-galactosidase) cDNA. This results in the expression of *lacZ* in the oocytes and the production of blue-colored β-galactosidase staining. (**B–D**) Labeling of only primordial follicles in adult mouse ovaries. Three-month-old *Sohlh1-CreER^T2^;R26R* females were given intraperitoneal injections of tamoxifen for 3 consecutive days. (B) The labeled primordial follicles (shown by the blue dots in the oocytes) can be seen 5 days after the injection. Arrows indicate the unlabeled growing follicles. (C) Labeled primordial follicles are still seen in ovaries 7 weeks after tamoxifen injection. (D) Labeled primordial follicles are still seen in the ovaries 14 months after tamoxifen injection.

To trace the *in vivo* development of cortical primordial follicles throughout adulthood, we generated another knock-in mouse model in which *CreER^T2^* was driven by the endogenous promoter of *Sohlh1* (Supplementary Material, Fig. S4A and B). The *Sohlh1* promoter is only active in the oocytes of primordial follicles (13), and by crossing these mice to a *Rosa26 reporter* (*R26R*) mouse line, we were able to exclusively label the oocytes of primordial follicles (Fig. 3A).

We injected the *Sohlh1-CreER^T2^;R26R* mice with tamoxifen at 3 months of age (80 mg/kg BW, ∼2 mg per mouse, daily for 3 consecutive days). Five days after the first tamoxifen injection, primordial follicles in the cortical region were specifically labeled as shown by the β-galactosidase staining in the oocytes (Fig. 3B). These labeled primordial follicles were always found in the ovaries as seen at 7 weeks (Fig. 3C) and at 14 months (the longest time studied) (Fig. 3D) after tamoxifen injection.

Notably, no oocytes within primary or further-developed follicles (Fig. 3B, arrows) were labeled 5 days after the first tamoxifen administration. This showed a stringent labeling specificity for primordial follicles that was in accordance with the reported *Sohlh1* promoter activity (13). Neither vehicle-injected *Sohlh1-CreER^T2^;R26R* mice (Supplementary Material, Fig. S5A) nor tamoxifen-injected *R26R* mice (Supplementary Material, Fig. S5B) had any labeled primordial follicles, and this confirmed the specificity of this inducible *Sohlh1-CreER^T2^* mouse model. In addition, the development of ovarian follicles was not influenced by tamoxifen injection at adulthood (see Supplementary Material, Text and Supplementary Material, Fig. S6 A and B).

We found that ∼30% of the primordial follicles in the adult mouse ovaries could be labeled by tamoxifen administration (Fig. 3B). This level of efficiency, however, did not affect our ability to trace the follicular development because we used sufficient numbers of experimental animals as described later.

### Tracing the developmental dynamics of primordial follicles in the adult mouse ovary

To understand the nature of primordial follicular development in adult life, we traced the *in vivo* development of labeled primordial follicles to different ages in the *Sohlh1-CreER^T2^;R26R* mice. The classification of follicles was based on well-established standards (14). For this work, a total of 140 mice were sacrificed between 5 and 57 days after the first tamoxifen injection at 3 months of age.

#### Tracing the development of adult primordial follicles to the primary stage

**Figure 4.**
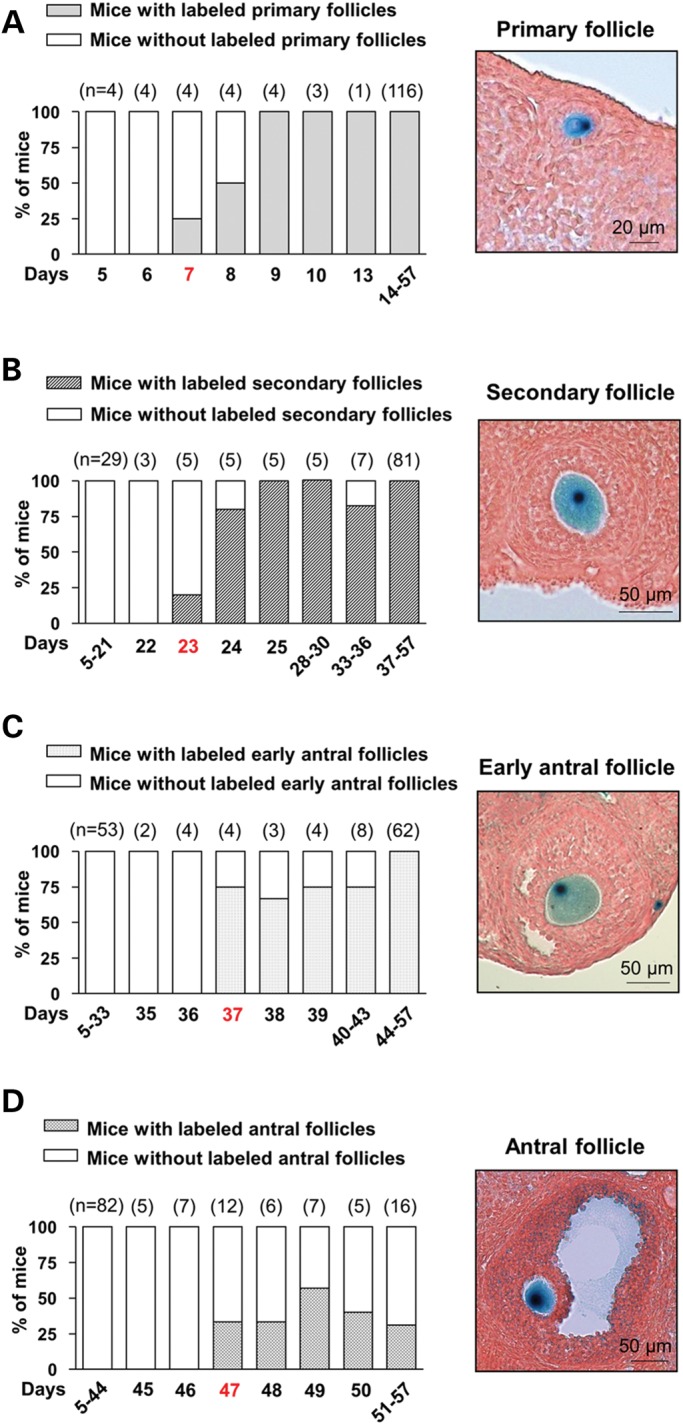
Tracing the development of primordial follicles in adult *Sohlh1-CreER^T2^;R26R* mice. *Sohlh1-CreER^T2^;R26R* adult females were injected with tamoxifen and sacrificed at different time points. The Y-axis is the percentage of mice showing labeled follicles. The numbers of mice used for each time point are shown above the bars. Representative labeled follicles are shown in the right panels. (**A**) A minimum of 7 days is needed for labeled primordial follicles to reach the primary stage. Labeled primary follicles can be found in all mice sacrificed from 9 to 57 days after tamoxifen injection. (**B**) A minimum of 23 days is needed for labeled primordial follicles to develop into secondary follicles. (**C**) A minimum of 37 days is needed for labeled primordial follicles to develop into the early antral stage. (**D**) A minimum of 47 days is needed for labeled primordial follicles to develop into the antral stage.

We first studied the transition from primordial to primary follicles. As shown in Figure 4A, labeled primary follicles were first observed in one out of four mice (25%) sacrificed 7 days after the first tamoxifen injection. Two out of four (50%) mice showed labeled primary follicles 8 days after tamoxifen injection, and from 9 days onward, labeled primary follicles were seen in all sacrificed mice (100%). We conclude, therefore, that it takes 7–9 days for primordial follicles to develop into primary stage follicles.

The fact that labeled primary follicles were always observed in all 124 mice sacrificed from 9 to 57 days after tamoxifen injection (Fig. 4A) indicated that the activation of primordial follicles might take longer than 9 days and that such activation occurs in a progressive manner as previously hypothesized (3,15).

#### Tracing of adult primordial follicles to the secondary stage

We next looked into how the primordial follicles reached the secondary stage. As shown in Figure 4B, labeled secondary follicles were seen in one out of five (20%) mice sacrificed 23 days after tamoxifen injection, four out of five (80%) mice sacrificed 24 days after tamoxifen injection, and in almost all mice sacrificed from 25 to 57 days after tamoxifen injection. These results indicated that a minimum of 23–24 days was needed for the primordial follicles to reach the secondary stage.

If 7 days are assumed to be the minimum time needed to develop from primordial to primary follicles, the shortest dwelling time of primary follicles in mouse ovaries is 16 days.

#### Tracing of adult primordial follicles to the early antral stage

As shown in Figure 4C, labeled early antral follicles were found in three out of four (75%) mice sacrificed 37 days after tamoxifen injection, 11 out of 15 (73%) mice sacrificed from 38 to 43 days after tamoxifen injection, and in all mice sacrificed from 44 to 57 days after tamoxifen injection. Thus, the development from primordial follicles to the early antral stage takes at least 37 days.

If a minimum of 23 days is assumed for primordial follicles to reach the secondary stage, the time for secondary follicles to develop into early antral follicles in mouse ovaries is at least 14 days.

#### Tracing of adult primordial follicles to the antral stage

No labeled antral follicles were seen in 87 mice sacrificed from 5 to 45 days after tamoxifen injection or in 7 mice sacrificed 46 days after tamoxifen injection (Fig. 4D). After that, a stable proportion of mice (30%–50%) sacrificed from 47 to 57 days after tamoxifen injection contained labeled antral follicles (Fig. 4D). Thus, it takes a minimum of 47 days for a primordial follicle to go through the different developmental stages and become an antral follicle in adult mouse ovaries.

The minimum dwelling time for an early antral follicle can be calculated as 10 days because it takes at least 37 days to develop from primordial to early antral follicles.

#### Comparison of the developmental dynamics of the postnatal and adult primordial follicles

The developmental dynamics of adult primordial follicles in the ovarian cortex are distinct from those of the first wave of activated primordial follicles in the ovarian medulla. We have summarized the age-dependent minimal times needed for their respective development in Table 1.

**Table 1.** The distinct age-dependent minimal developmental times of the two classes of primordial follicles

	Primordial to primary	Primordial to secondary	Primordial to early antral	Primordial to antral
First wave of activated primordial follicles	Right after formation	8 days	13 days	23 days
Adult cortical primordial follicles	7–9 days	23–24 days	37 days	47 days

### Long-term tracing of primordial follicles

**Figure 5.**
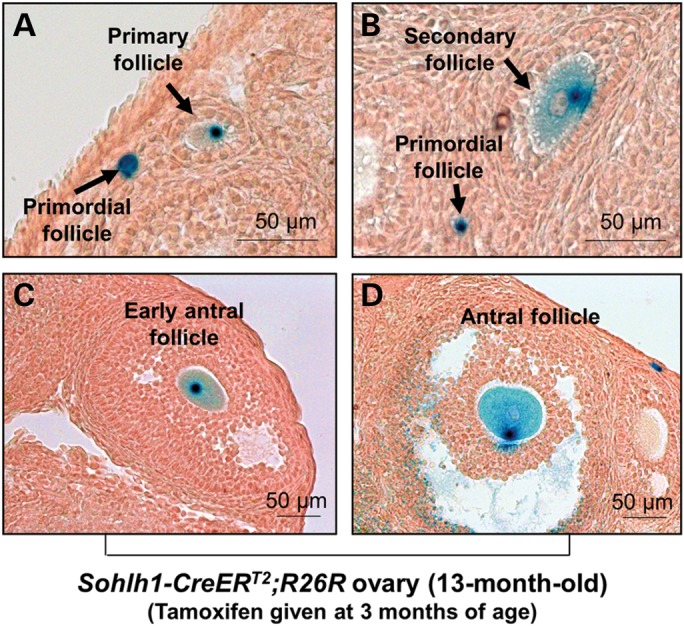
Labeled primordial follicles persisted to the end of the reproductive lifespan in *Sohlh1-CreER^T2^;R26R* mouse ovaries. *Sohlh1-CreER^T2^;R26R* adult females were given tamoxifen and sacrificed 10 months later. (**A**) A representative labeled primary follicle with a labeled primordial follicle in the same field. (**B**) A representative labeled secondary follicle with a labeled primordial follicle in the same field. (**C**) A representative labeled early antral follicle. (**D**) A representative labeled antral follicle.

To trace the development of primordial follicles throughout the entire mouse reproductive lifespan, we labeled the adult cortical primordial follicles by administering tamoxifen to 17 *Sohlh1-CreER^T2^;R26R* mice at 3 months of age and sacrificed the mice over a period of 2 to 14 months after tamoxifen injection. Labeled primordial, primary, secondary and early antral follicles were always observed in all of the mice sacrificed at various times during this period (Fig. 3D and Fig. 5A–C). In addition, labeled antral follicles were found in mice sacrificed 10 months after tamoxifen injection (13 months old) (Fig. 5D).

This result indicated that the primordial follicles labeled in early adulthood were competent for activation after a long period of dormancy and could contribute to ovulation. This finding is in accordance with the notion that a fixed pool of primordial follicles serves as the only source of developing follicles throughout the reproductive lifespan of mice (16–18).

## DISCUSSION

In the mammalian ovary, progressive activation of primordial follicles serves as the source of fertilizable ova. However, very little is known about the developmental dynamics of primordial follicles under physiological conditions. In this study, we have generated tamoxifen-inducible *Foxl2-CreER^T2^* and *Sohlh1-CreER^T2^* knock-in mouse models to specifically label the first wave of postnatally activated primordial follicles and the adult cortical primordial follicles, respectively.

We showed that the first wave of activated primordial follicles remain in the mouse ovaries for ∼3 months after birth and contribute to the onset of puberty and to fertility during early adulthood. During this period, primordial follicles that are formed after birth and located in the ovarian cortex gradually replace the first wave of follicles until they become the sole source of follicles from 3 months until the end of the reproductive life. We have also determined the age-dependent time periods required for the two classes of primordial follicles to develop into advanced stages and have shown that the developmental dynamics were completely different for the two types of primordial follicles (Table 1). Moreover, our follicular tracing data showed that primordial follicles that are labeled early in life can persist in the ovaries until the end of reproductive life and that these follicles were competent to develop into antral stage.

The visual *in vivo* tracing of follicles performed in this work provides a precise picture of long-term follicular development starting from the primordial stage. Such accurate tracing has not been possible by autoradiographic labeling of follicles (19,20). We believe that our results provide a relatively conclusive picture of how the two distinct classes of primordial follicles develop under normal physiological conditions. Such an understanding is valuable for studying ovarian organogenesis and physiology as well as ovarian diseases that are related to altered follicular development.

It has been generally believed that the first wave of activated follicles are anovulatory (5,7) even though oocytes from the first wave of follicles can mature *in vitro* and generate live pups (21,22). Our data clearly show, however, that the first wave of follicles plays an active role in providing mature eggs until ∼3 months of age. Thus, it is likely that the fast-growing first wave of follicles facilitates the establishment of the hypothalamic–pituitary–ovarian axis and thereby plays a key role in the onset of puberty and the initiation of reproductive life.

Given the fact that the growth pattern of the first wave of activated follicles is conserved among mammals (including humans) (23,24), it can be postulated that the fertility of women from puberty onset to young adulthood might rely on the first wave of follicles that are already activated at the fetal stage. In contrast to this first wave of activated follicles, we have shown that the relatively slow-growing adult primordial follicles contribute to continuous ovulation throughout the middle and late stages of reproductive life, and the gradual but steady loss of the cortical primordial follicles leads to reproductive aging. It is possible that the developmental dynamics of adult primordial follicles in mice might be similar to adult primordial follicle development in humans.

Taken together, this study presents several lines of *in vivo* evidence that clarify the distinct developmental dynamics of two classes of primordial follicles under physiological conditions and provides a solid foundation for studying how primordial follicles develop in humans.

## MATERIALS AND METHODS

### Reagents

Restriction enzymes were purchased from New England Biolabs, UK. Antibiotics were purchased from Invitrogen, US. Tamoxifen and corn oil were purchased from Sigma-Aldrich, Germany. Reagents for β-galactosidase staining [paraformaldehyde, magnesium chloride, ethylene glycol tetraacetic acid, sodium deoxycholate, NP40, potassium ferricyanide, potassium ferrocyanide, 5-Bromo-4-chloro-3-indolyl β-D-galactoside (X-gal) and 0.1% nuclear fast red solution] were all purchased from Sigma–Aldrich. Paraffin and hematoxylin were purchased from Histolab, Sweden.

### Generation of a *Foxl2-CreER^T2^* knock-in mouse model to label the first postnatal wave of activated follicles

The mouse *Foxl2* gene contains one exon, and we aimed to keep the original open reading frame of *Foxl2* intact. An ∼8 kb region used to construct the targeting vector was first sub-cloned from a C57BL/6 BAC clone that contained the full-length *Foxl2* allele (RP23: 142D12). As shown in Supplementary Material, Figure S1A, the long arm extended 5.5 kb from the 5′ end to the site of the cassette insertion after the coding region of *Foxl2*. The short arm extended 2.5 kb from the site of the cassette insertion to the 3′ end. An *IRES-CreER^T2^-FRT-Neo-FRT* cassette was inserted at 259 bp downstream of the STOP codon of *Foxl2* using Red/ET recombineering technology (Gene Bridges, Germany).

The targeting construct was sub-cloned into a backbone vector pSP72 (Promega, US) containing an ampicillin selection cassette for re-transformation of the construct prior to electroporation. Ten micrograms of the targeting vector were linearized by NotI and then transfected by electroporation of BA1 (C57BL/6 × 129/SvEv) (Hybrid) embryonic stem (ES) cells. After selection with G418 antibiotic, surviving clones were selected by PCR and Southern blot analysis following standard procedures (Supplementary Material, Fig. S1B). The positive clone was microinjected into C57BL/6 blastocysts following standard procedures. Resulting chimeric mice with a high percentage agouti coat color were mated to wild-type C57BL/6 mice to generate F1 heterozygous offspring. The heterozygous mice were healthy and fertile. The neomycin expression cassette was excised *in vivo* by crossing F1 *Foxl2-CreER^T2^* mice with transgenic mice expressing flippase (FLP) (003800, Jackson Laboratory, US). *Foxl2-CreER^T2^* males were mated with *mT/mG* females (007576, Jackson Laboratory) to generate *Foxl2-CreER^T2^;mT/mG* mice.

### Generation of *Sohlh1-CreER^T2^* knock-in mice to label primordial follicles in adulthood

A ∼13 kb region used to construct the targeting vector was first sub-cloned from a positively identified C57BL/6 (RP23: 57O11) BAC clone that contained the full-length *Sohlh1* allele. The region was designed such that the short arm extended 2.3 kb 5′ to the ATG of exon 1. The long arm was located on the 3′ side of exon 8 and was 6.5 kb long. The *CreER^T2^-FRT-Neo-FRT* cassette replaced 4.2 kb of the gene, including exons 1–8 at the ATG site (Supplementary Material, Fig. S4A).

The targeting construct was sub-cloned into a ∼2.4 kb pSP72 backbone vector. Ten micrograms of targeting vector were linearized by SwaI. The electroporation, ES cell characterization (Supplementary Material, Fig. S4B), blastocyst injection and neomycin cassette removal were performed with standard procedures as described earlier. The heterozygous mice were found to be healthy and fertile. *Sohlh1-CreER^T2^* males were mated with *R26R* females (003474, Jackson Laboratory) to generate the *Sohlh1-CreER^T2^;R26R* mice.

All mice were crossed with C57BL/6 mice (Charles River, US) for six generations to obtain identical genetic backgrounds. Mice were housed in controlled environmental conditions with free access to water and food. Illumination was on between 0600 and 1800. Experimental protocols were approved by the regional ethical committee of the University of Gothenburg, Sweden.

### Tamoxifen administration in *Foxl2-CreER^T2^;mT/mG* mice

Tamoxifen was resuspended at 100 mg/mL in 95% ethanol and further diluted with corn oil to a final concentration of 20 mg/mL (25). For embryonic injection, *mT/mG* females were first plugged by *Foxl2-CreER^T2^* males. A single intraperitoneal injection of tamoxifen (15 mg/kg BW, ∼600 µg per mouse) was given to pregnant females at E16.5 (the day the vaginal plug appeared was counted as E0.5). Because the tamoxifen injection compromised the ability of the mice to have spontaneous vaginal delivery, pups were delivered by Cesarean section at E20. Surrogate mothers were used to nurture the pups. For postnatal injection, *Foxl2-CreER^T2^;mT/mG* females were given a single intraperitoneal injection of tamoxifen at PD5 at a dose of 15 mg/kg BW.

### Histological analysis and quantification of ovarian follicles from *Foxl2-CreER^T2^;mT/mG* mice

To observe the ovarian morphology of *Foxl2-CreER^T2^;mT/mG* mice, ovaries were fixed in 4% paraformaldehyde, dehydrated and embedded in paraffin. The paraffin-embedded ovaries were serially cut into 8-μm sections and rehydrated. The fluorescent images were taken with a Zeiss Axio Scope A1 upright microscope installed with filter sets for mT (tdTomato, 554/581 nm) and mG (EGFP, 488/507 nm) and merged with the Zeiss AxioVision software.

For quantification of follicles, the fluorescent images from every five sections per ovary were used to count the follicles. The classification of follicular development was based on well-established standards by Pedersen and Peters (14). The numbers of growing follicles with green (designated as labeled) or red (designated as unlabeled) granulosa cells in each image were counted.

### Tamoxifen administration in *Sohlh1-CreER^T2^;R26R* mice

To reach the optimal labeling efficiency, 3-month-old adult *Sohlh1-CreER^T2^;R26R* female mice were given an intraperitoneal injection of 80 mg/kg BW (∼2 mg per mouse) tamoxifen per day for three consecutive days. The CreER^T2^-mediated DNA recombination can take place within 24 h after tamoxifen injection (26,27), so the day of the first tamoxifen injection was counted as day 0. The labeled primordial follicles can be clearly visualized by β-galactosidase staining 5 days after the first tamoxifen injection. For tracing the follicular development, 140 mice were sacrificed between 5 and 57 days after the first tamoxifen injection. For long-term tracing of primordial follicles, 17 mice were sacrificed between 2 to 14 months after the first tamoxifen injection.

### β-galactosidase staining of *Sohlh1-CreER^T2^;R26R* ovaries

Whole-mount β-galactosidase staining was performed to visualize the labeled follicles in the ovaries of *Sohlh1-CreER^T2^;R26R* mice. Briefly, ovaries were fixed in 4% paraformaldehyde for 1 h at 4°C and then rinsed 3 times for 15 min each in a buffer consisting of 1 × PBS (pH 7.4), 2 mM magnesium chloride, 5 mM ethylene glycol tetraacetic acid, 0.01% sodium deoxycholate and 0.02% NP40 at room temperature. The ovaries were then incubated with a staining solution consisting of 1 × PBS (pH 7.4), 2 mM magnesium chloride, 0.01% sodium deoxycholate, 0.02% NP40, 5 mM potassium ferricyanide, 5 mM potassium ferrocyanide and 1 mg/mL X-gal at 37°C overnight.

Following staining, the ovaries were re-fixed in 4% paraformaldehyde for 8 h, embedded in paraffin, serially cut into 8-μm sections and counterstained with 0.1% nuclear fast red solution. The sections were examined under a Zeiss Axio Scope A1 upright microscope. A follicle was considered to be labeled if a blue dot was observed in the cytoplasm of the oocyte.

### Quantification of ovarian follicles for evaluating the influence of tamoxifen on follicular development

For quantification of ovarian follicles and the morphological studies shown in Supplementary Material, Figs S3 and S6, ovaries were fixed in 4% paraformaldehyde, dehydrated and embedded in paraffin. Paraffin-embedded ovaries were serially cut into 8-µm sections and stained with hematoxylin for morphological observation. Ovarian follicles at different stages of development, including primordial (type 2), primary (type 3), secondary (type 4 and 5), early antral (type 6) and antral (type 7) follicles were counted in all sections of an ovary based on the well-accepted standards established by Pedersen and Peters (14). Follicles that contained oocytes with clearly visible nuclei were scored in each section, as previously reported (28). Judged from careful morphological analysis, the incidence of counting the same follicle twice or of missing a follicle was low.

### Statistical analysis

All experiments were repeated at least 3 times. In Supplementary Material, Figures S3 and S6, the differences between the numbers of ovarian follicles in tamoxifen- and vehicle-injected mice were calculated with Student's *t*-test, and the difference was considered to be significant if *P* < 0.05.

## SUPPLEMENTARY MATERIAL

Supplementary Material is available at HMG online.

*Conflict of Interest statement*. None declared.

## FUNDING

This work was supported by grants (to K. L.) from the Young Researcher Award (Umeå University), the Jane and Dan Olssons Foundation, the LUA/ALF-medel Västra Götalandsregionen, AFA Insurance, the Swedish Research Council, the Swedish Cancer Foundation, the Faculty of Natural Science of the University of Gothenburg and the Novo Nordisk Foundation (Denmark). Funding to pay the Open Access publication charges for this article was provided by the Swedish Research Council.

## Supplementary Material

Supplementary Data

## References

[1] Eppig J.J., Bivens C.M., Viveiros M.M., de la Fuente R., Peter C.K.L., Eli Y.A. (2003). Regulation of mammalian oocyte maturation. The Ovary.

[2] Matzuk M.M., Burns K.H., Viveiros M.M., Eppig J.J. (2002). Intercellular communication in the mammalian ovary: oocytes carry the conversation. Science.

[3] Adhikari D., Liu K. (2009). Molecular mechanisms underlying the activation of mammalian primordial follicles. Endocr. Rev..

[4] Reddy P., Zheng W., Liu K. (2010). Mechanisms maintaining the dormancy and survival of mammalian primordial follicles. Trends Endocrinol. Metab..

[5] Hirshfield A.N. (1992). Heterogeneity of cell populations that contribute to the formation of primordial follicles in rats. Biol. Reprod..

[6] Mork L., Maatouk D.M., McMahon J.A., Guo J.J., Zhang P., McMahon A.P., Capel B. (2012). Temporal differences in granulosa cell specification in the ovary reflect distinct follicle fates in mice. Biol. Reprod..

[7] Eppig J.J., Handel M.A. (2012). Origins of granulosa cells clarified and complexified by waves. Biol. Reprod..

[8] Hirshfield A.N., DeSanti A.M. (1995). Patterns of ovarian cell proliferation in rats during the embryonic period and the first three weeks postpartum. Biol. Reprod..

[9] Peters H. (1969). The development of the mouse ovary from birth to maturity. Acta. Endocrinol. (Copenh).

[10] Schmidt D., Ovitt C.E., Anlag K., Fehsenfeld S., Gredsted L., Treier A.C., Treier M. (2004). The murine winged-helix transcription factor Foxl2 is required for granulosa cell differentiation and ovary maintenance. Development.

[11] Uda M., Ottolenghi C., Crisponi L., Garcia J.E., Deiana M., Kimber W., Forabosco A., Cao A., Schlessinger D., Pilia G. (2004). Foxl2 disruption causes mouse ovarian failure by pervasive blockage of follicle development. Hum. Mol. Genet..

[12] Muzumdar M.D., Tasic B., Miyamichi K., Li L., Luo L. (2007). A global double-fluorescent Cre reporter mouse. Genesis.

[13] Pangas S.A., Choi Y., Ballow D.J., Zhao Y., Westphal H., Matzuk M.M., Rajkovic A. (2006). Oogenesis requires germ cell-specific transcriptional regulators Sohlh1 and Lhx8. Proc. Natl. Acad. Sci. USA.

[14] Pedersen T., Peters H. (1968). Proposal for a classification of oocytes and follicles in the mouse ovary. J. Reprod. Fertil..

[15] McGee E.A., Hsueh A.J. (2000). Initial and cyclic recruitment of ovarian follicles. Endocr. Rev..

[16] Zuckerman S. (1951). The number of oocytes in the mature ovary. Recent Prog. Horm. Res..

[17] Lei L., Spradling A.C. (2013). Female mice lack adult germ-line stem cells but sustain oogenesis using stable primordial follicles. Proc. Natl. Acad. Sci. USA.

[18] Zhang H., Zheng W., Shen Y., Adhikari D., Ueno H., Liu K. (2012). Experimental evidence showing that no mitotically active female germline progenitors exist in postnatal mouse ovaries. Proc. Natl. Acad. Sci. USA.

[19] Oakberg E.F. (1979). Timing of oocyte maturation in the mouse and its relevance to radiation-induced cell killing and mutational sensitivity. Mutat. Res..

[20] Pedersen T. (1970). Follicle kinetics in the ovary of the cyclic mouse. Acta. Endocrinol. (Copenh).

[21] Eppig J.J., O'Brien M.J., Wigglesworth K., Nicholson A., Zhang W., King B.A. (2009). Effect of in vitro maturation of mouse oocytes on the health and lifespan of adult offspring. Hum. Reprod..

[22] O'Brien M.J., Pendola J.K., Eppig J.J. (2003). A revised protocol for in vitro development of mouse oocytes from primordial follicles dramatically improves their developmental competence. Biol. Reprod..

[23] Peters H., Byskov A.G., Himelstein-Braw R., Faber M. (1975). Follicular growth: the basic event in the mouse and human ovary. J. Reprod. Fertil..

[24] Lintern-Moore S., Peters H., Moore G.P., Faber M. (1974). Follicular development in the infant human ovary. J. Reprod. Fertil..

[25] John G.B., Gallardo T.D., Shirley L.J., Castrillon D.H. (2008). Foxo3 is a PI3K-dependent molecular switch controlling the initiation of oocyte growth. Dev. Biol..

[26] Hayashi S., McMahon A.P. (2002). Efficient recombination in diverse tissues by a tamoxifen-inducible form of Cre: a tool for temporally regulated gene activation/inactivation in the mouse. Dev. Biol..

[27] Feil R., Brocard J., Mascrez B., LeMeur M., Metzger D., Chambon P. (1996). Ligand-activated site-specific recombination in mice. Proc. Natl. Acad. Sci. USA.

[28] Reddy P., Liu L., Adhikari D., Jagarlamudi K., Rajareddy S., Shen Y., Du C., Tang W., Hämäläinen T., Peng S.L. (2008). Oocyte-specific deletion of Pten causes premature activation of the primordial follicle pool. Science.

