# Supplementary material for: Two classes of ovarian primordial follicles exhibit distinct developmental dynamics and physiological functions
Source: Hum Mol Genet. 2013 Oct 1;23(4):920–8. doi: 10.1093/hmg/ddt486 (PMC3900105; doi:10.1093/hmg/ddt486)
Supplement: Supplementary Data [file supp_23_4_920__index.html]

Two classes of ovarian primordial follicles exhibit distinct developmental dynamics and physiological functions — Two classes of ovarian primordial follicles exhibit distinct developmental dynamics and physiological functions — Two classes of ovarian primordial follicles exhibit distinct developmental dynamics and physiological functions — Supplementary Data 

# Two classes of ovarian primordial follicles exhibit distinct developmental dynamics and physiological functions

## Supplementary Data

Supplementary Data

**Files in this Data Supplement:**

- Supplementary Figures - pdf file
- Supplementary Data - Docx file
