# Supplementary material for: Two classes of ovarian primordial follicles exhibit distinct developmental dynamics and physiological functions
Source: Hum Mol Genet. 2013 Oct 1;23(4):920–8. doi: 10.1093/hmg/ddt486 (PMC3900105; doi:10.1093/hmg/ddt486)
Supplement: Supplementary Data [file supp_ddt486_ddt486supp_figs.pdf]

Fig. S1

A

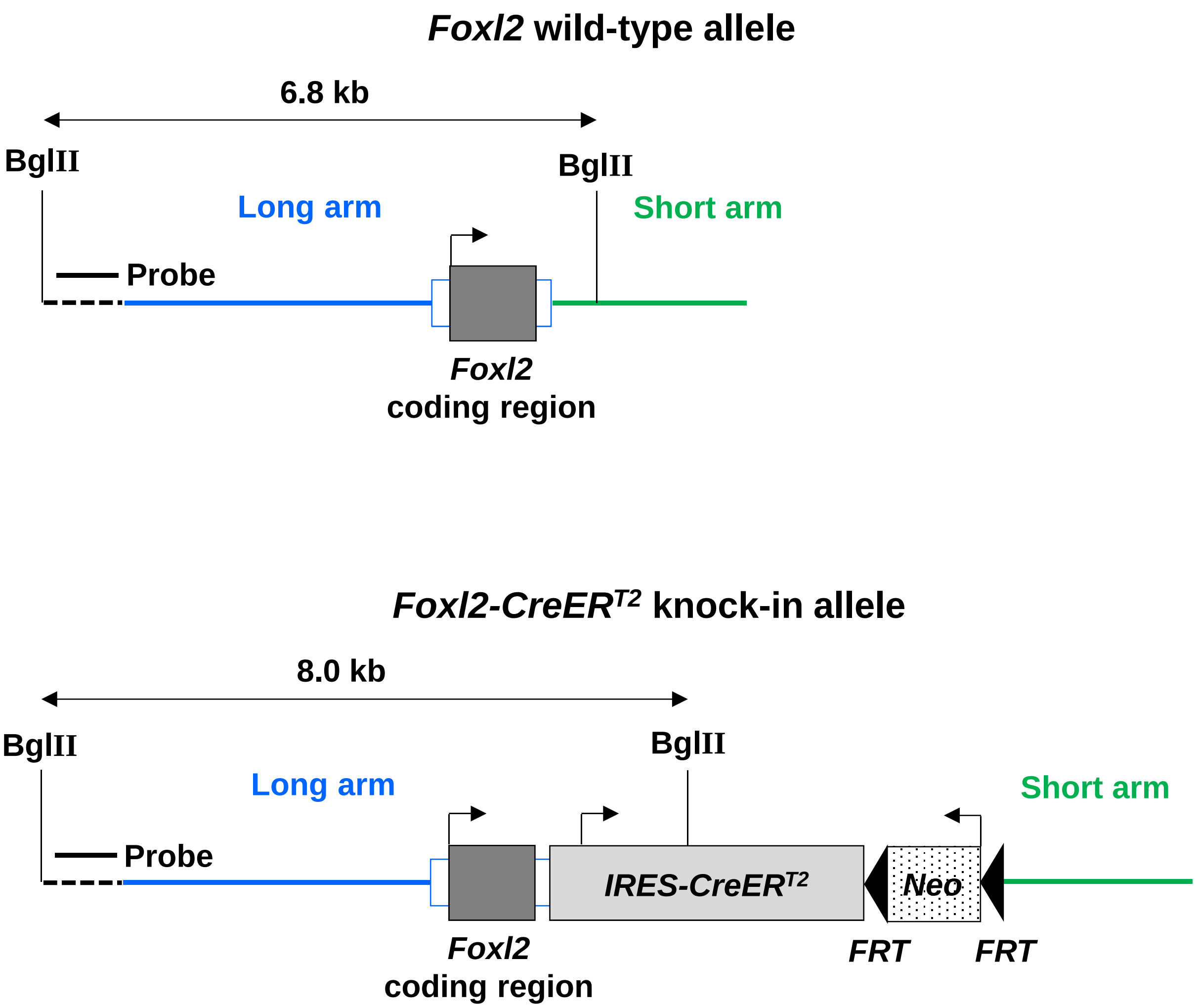

Foxl2 wild-type allele
6.8 kb
BglII
BglII
Long arm
Short arm
Probe
Foxl2
coding region
Foxl2-CreER^T2 knock-in allele
8.0 kb
BglII
BglII
Long arm
Short arm
Probe
IRES-CreER^T2
Neo
Foxl2
coding region
FRT
FRT

B

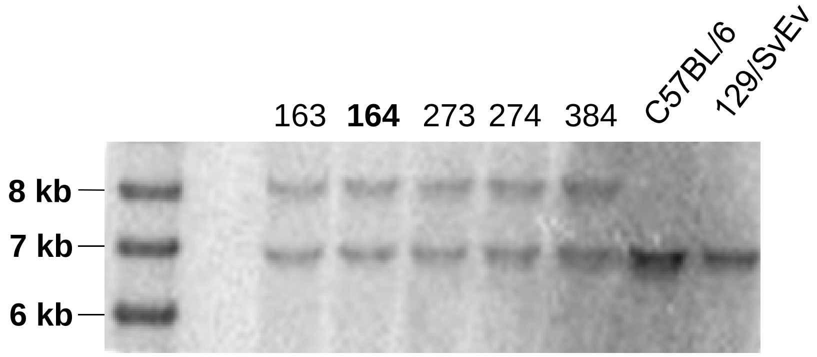

163
164
273
274
384
C57BL/6
129/SvEv
8 kb
7 kb
6 kb

Fig. S1. Construction of *Foxl2-CreER*^{T2} mice.

(**A**) The schematic structures of the mouse *Foxl2* wild-type and knock-in alleles. The *IRES-CreER*^{T2}*-FRT-Neo-FRT* cassette is inserted behind the *Foxl2* coding region so that the expression of *Foxl2* is not affected.

(**B**) Southern blotting screening for positive ES clones. Clone 164 showed an 8.0 kb BglII band and was used for blastocyst injection. Genomic DNAs from C57BL/6 and 129/SvEv mice were used as negative controls.

Fig. S2

A

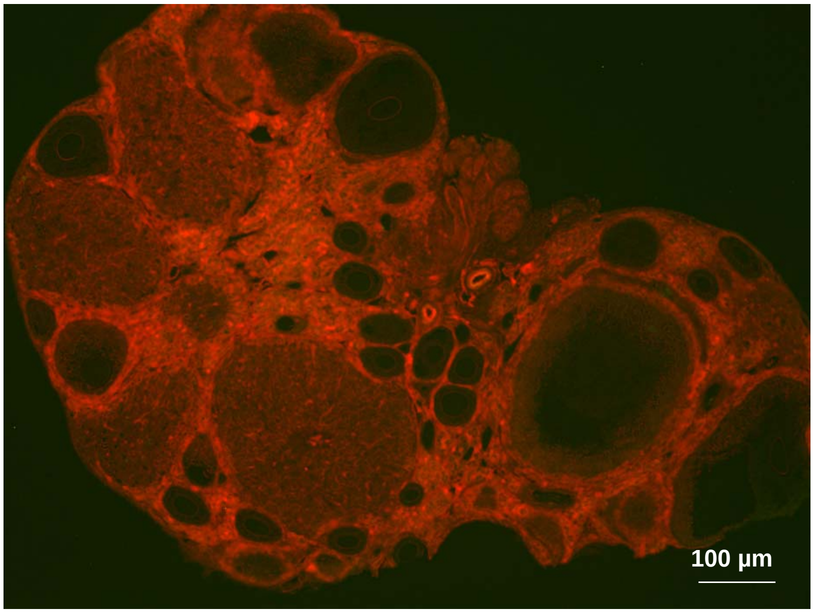

Foxl2-CreER^{T2};mT/mG **ovary (PD45)**
(Vehicle given at PD5)

B

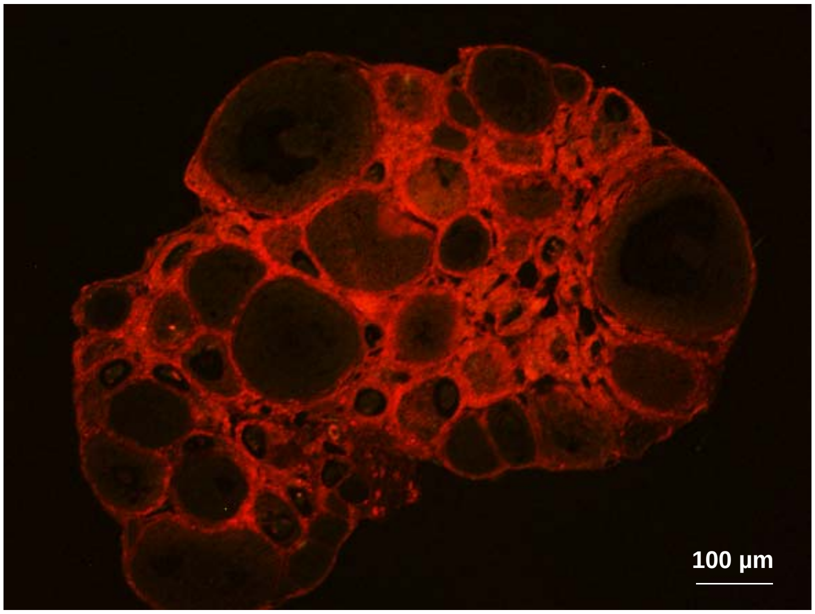

mT/mG **ovary (PD45)**
(Tamoxifen given at PD5)

Fig. S2. Negative controls for tamoxifen-induced CreER^{T2} recombinase activity in the *Foxl2-CreER^{T2}* mice.

(**A**) The *Foxl2-CreER^{T2};mT/mG* pups were given a single intraperitoneal injection of vehicle at PD5, and their ovaries were analyzed at PD45. No follicles were labeled with green fluorescence indicating that there was no leakage of CreER^{T2} recombinase activity without tamoxifen injection. (**B**) The *mT/mG* pups were given a single intraperitoneal injection of tamoxifen (15 mg/kg BW) at PD5, and their ovaries were analyzed at PD45. No follicles were labeled with green fluorescence indicating that the green fluorescence (mG) cannot be switched on by tamoxifen in the absence of the *CreER^{T2}* cassette.

Fig. S3

A

Quantification of ovarian follicles at PD8
(Tamoxifen or vehicle to E16.5 mothers)

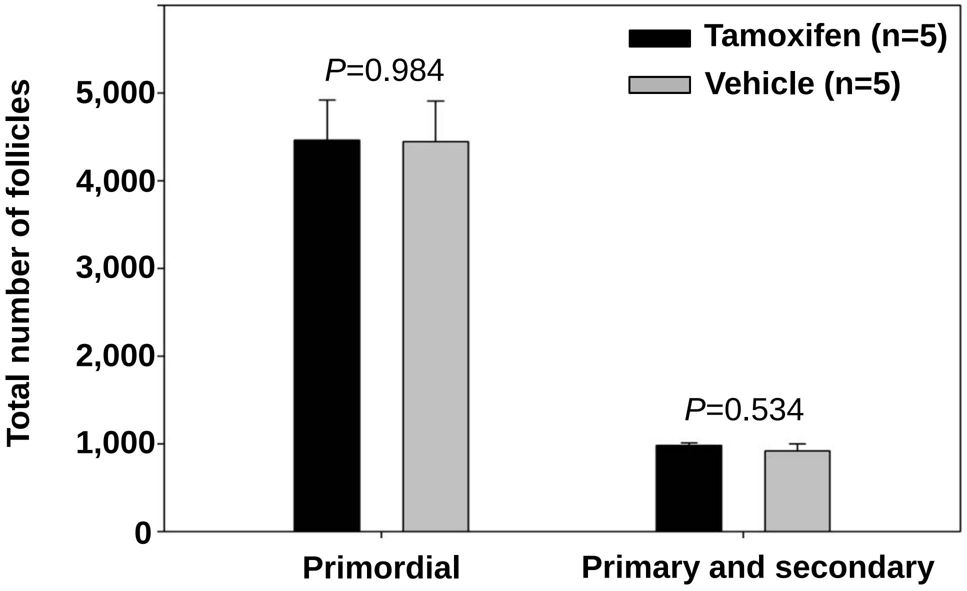

B

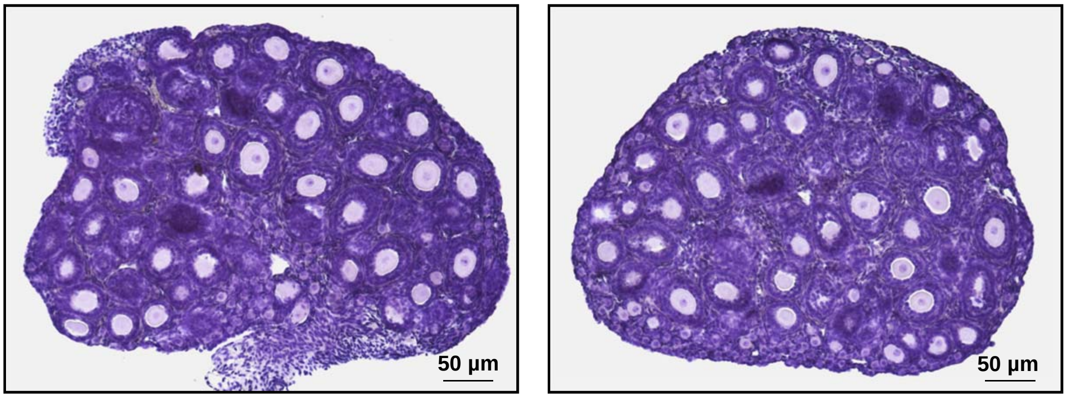

PD8 ovary
(Tamoxifen to E16.5 mothers)

PD8 ovary
(Vehicle to E16.5 mothers)

C

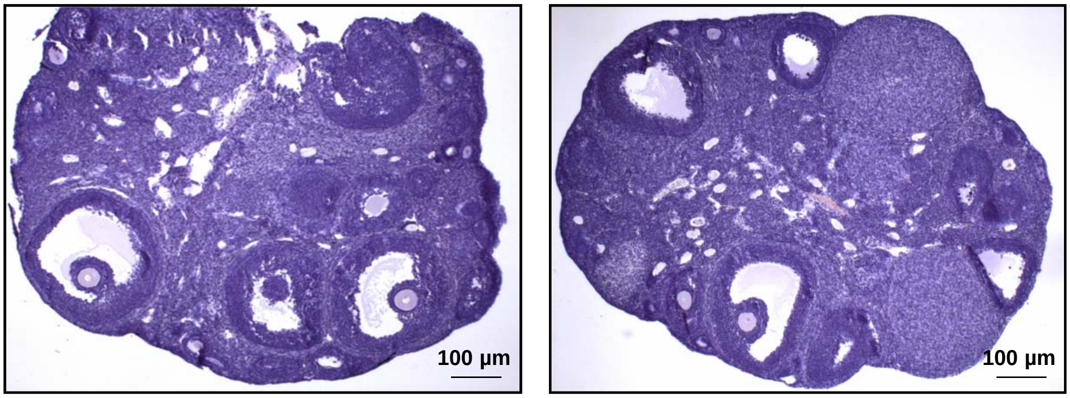

Two-month-old ovary
(Tamoxifen to E16.5 mothers)

Two-month-old ovary
(Vehicle to E16.5 mothers)

Fig. S3. The formation and development of ovarian follicles were not affected by embryonic injection of tamoxifen.

Pregnant *mT/mG* females were given a single intraperitoneal injection of vehicle or tamoxifen (15 mg/kg BW) at E16.5.

(**A**) At PD8, primordial and growing (primary and secondary) follicles were quantified in all sections per ovary based on the well-accepted standards. No significant difference in the numbers of primordial or growing follicles was seen between pups from tamoxifen- and vehicle-injected mothers. Values are means ± S.E.M. The *P* values are shown above the bars.

(**B** and **C**) The gross ovarian morphology of pups from tamoxifen-injected mothers was compared to that of pups from vehicle-injected mothers, and no difference was found at PD8 (B) or 2 months of age (C).

Fig. S4

A

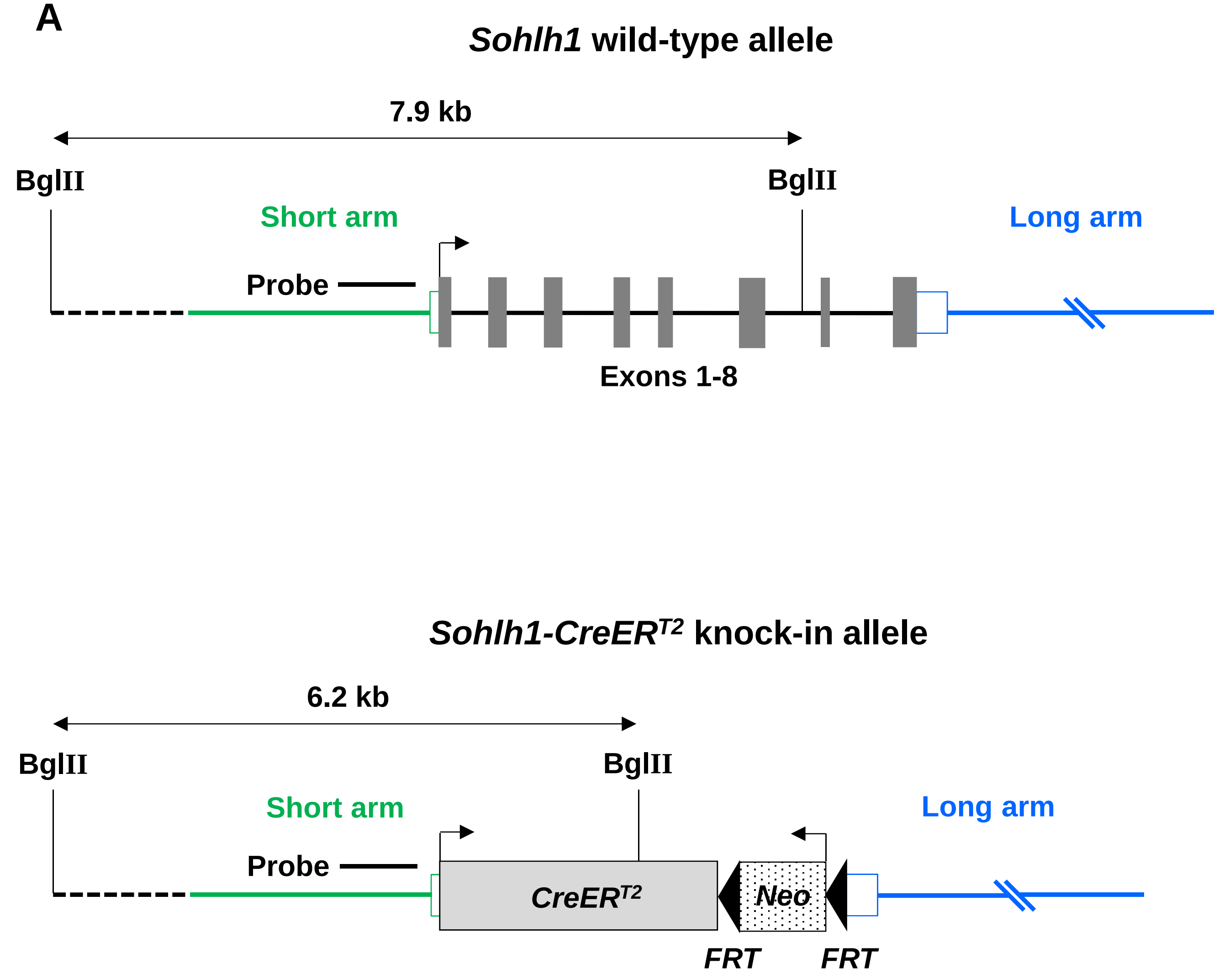

Sohlh1 wild-type allele
7.9 kb
BglII
BglII
Short arm
Long arm
Probe
Exons 1-8
Sohlh1-CreERT2 knock-in allele
6.2 kb
BglII
BglII
Short arm
Long arm
Probe
CreERT2
Neo
FRT
FRT

B

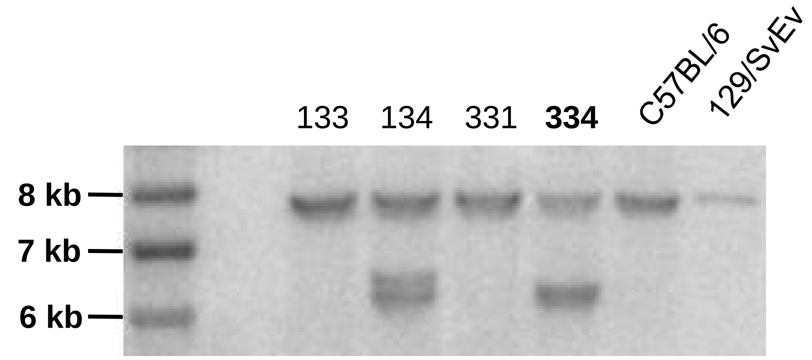

133
134
331
334
C57BL/6
129/SvEv
8 kb
7 kb
6 kb

Fig. S4. Construction of *Sohlh1-CreER*^{T2} mice.

(**A**) The schematic structures of the mouse *Sohlh1* wild-type and knock-in alleles. The *CreER*^{T2}-*FRT-Neo-FRT* cassette is inserted immediately after the ATG translational start and replaces exons 1 to 8.

(**B**) Southern blotting screening for positive ES clones. Clone 334 showed the 6.2 kb BglII band and was used for blastocyst injection. Genomic DNAs from C57BL/6 and 129/SvEv mice were used as negative controls.

Fig. S5

A

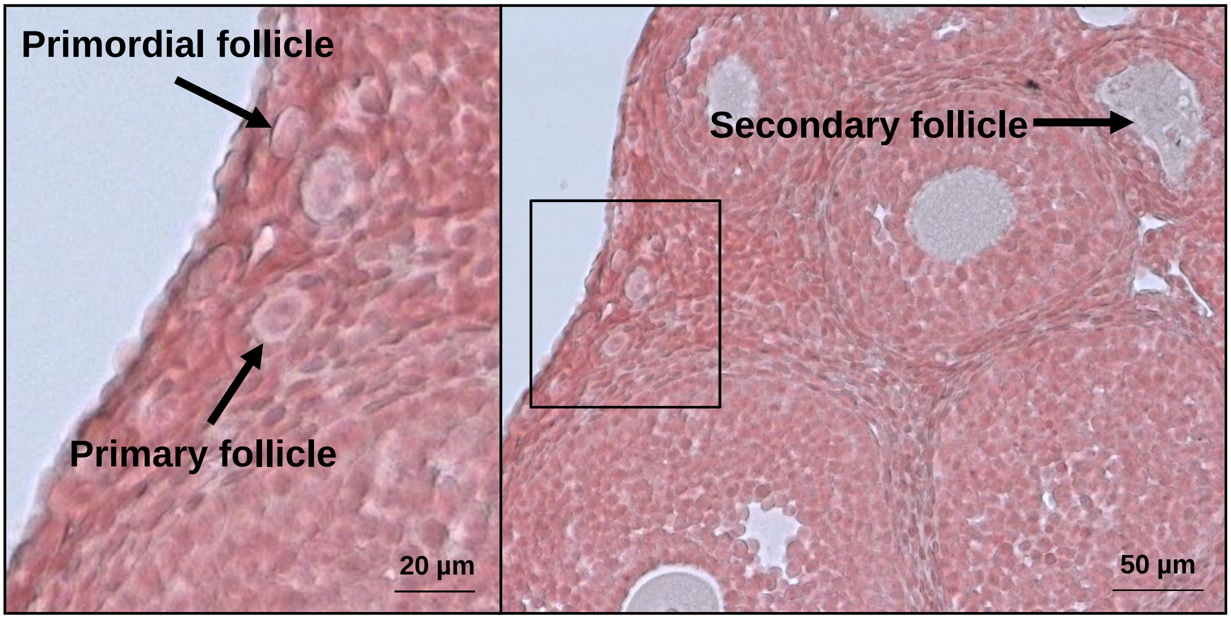

Sohlh1-CreER^{T2}*;R26R* ovary (4-month-old)
(Vehicle given at 3 months of age)

B

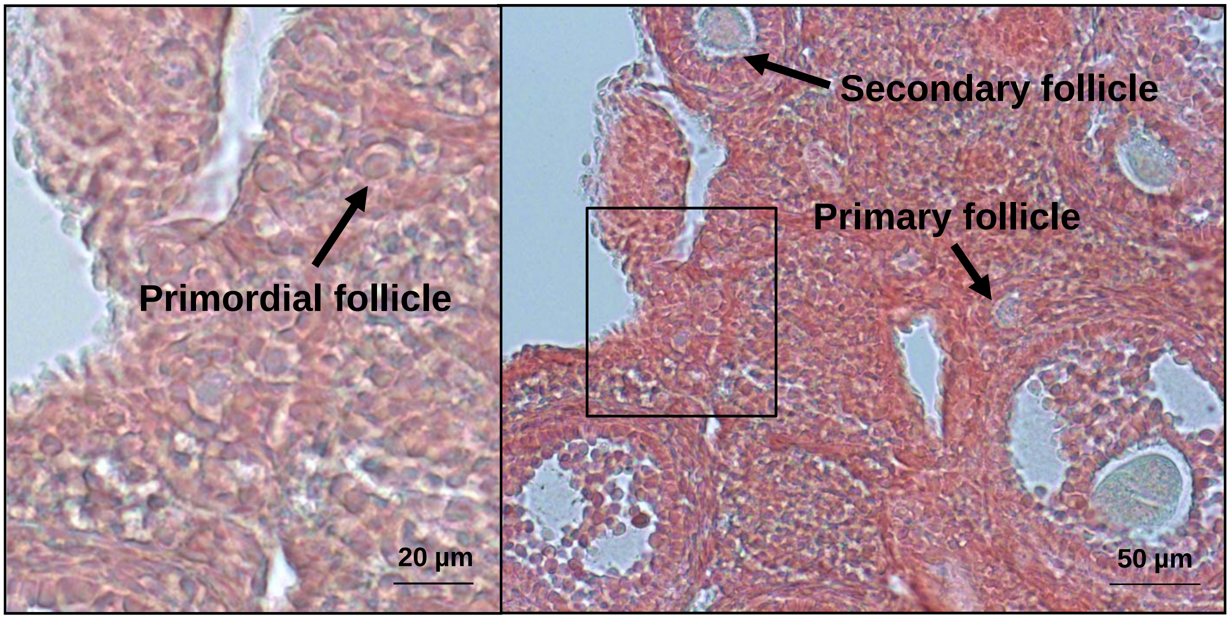

R26R ovary (4-month-old)
(Tamoxifen given at 3 months of age)

Fig. S5. Negative controls for tamoxifen-induced CreER^{T2} recombinase activity in *Sohlh1-CreER^{T2}* mice.

(**A**) Three-month-old *Sohlh1-CreER^{T2};R26R* female mice were given intraperitoneal injections of vehicle for 3 consecutive days. One month after the injection, their ovaries were subjected to β-galactosidase staining and analyzed. No oocytes showed positive blue β-galactosidase staining indicating that there is no leakage of CreER^{T2} recombinase activity without tamoxifen injection.

(**B**) Three-month-old *R26R* females were given daily intraperitoneal injections of tamoxifen (80 mg/kg BW) for 3 consecutive days. One month after the injection, the ovaries were subjected to β-galactosidase staining and analyzed. No oocytes showed positive blue β-galactosidase staining indicating that the expression of β-galactosidase cannot be switched on by tamoxifen in the absence of the *CreER^{T2}* cassette. Representative follicles at various developmental stages are indicated by arrows.

Fig. S6

A

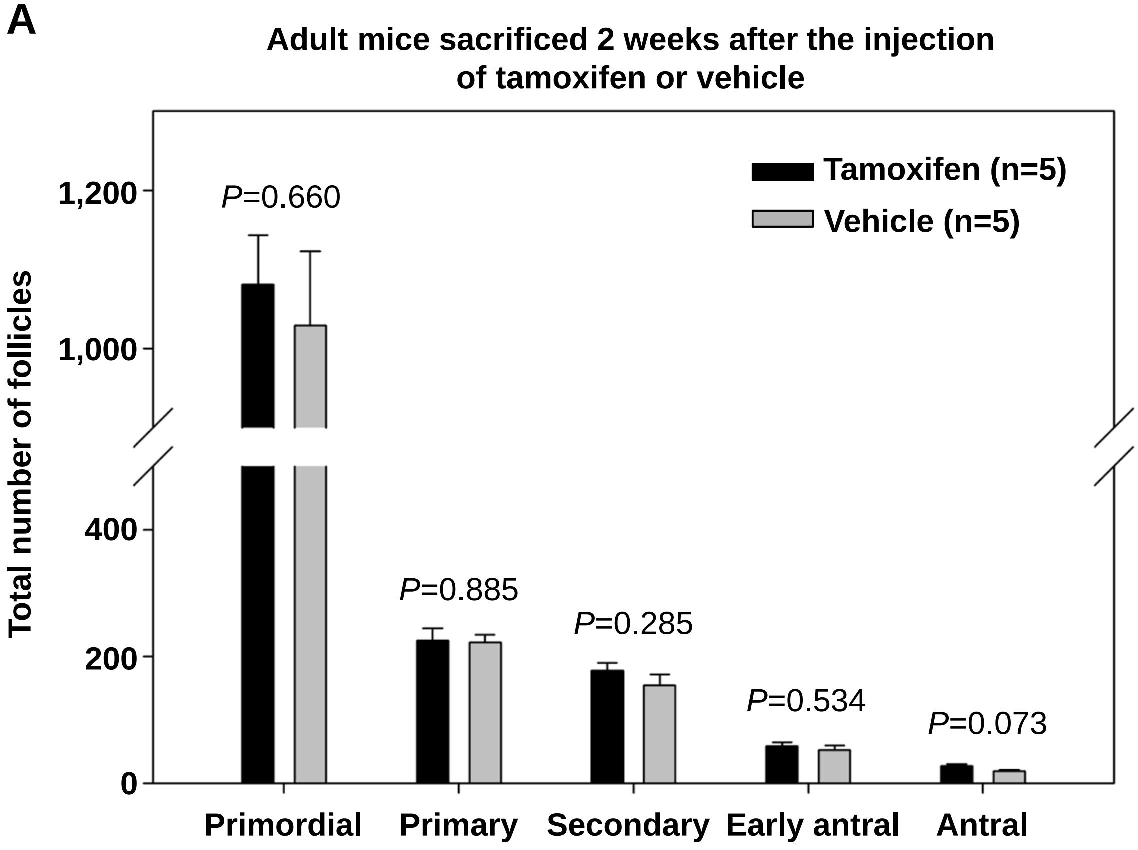
Adult mice sacrificed 2 weeks after the injection
of tamoxifen or vehicle
Tamoxifen (n=5)
Vehicle (n=5)
Total number of follicles
1,200
1,000
400
200
0
P=0.660
P=0.885
P=0.285
P=0.534
P=0.073
Primordial
Primary
Secondary
Early antral
Antral

B

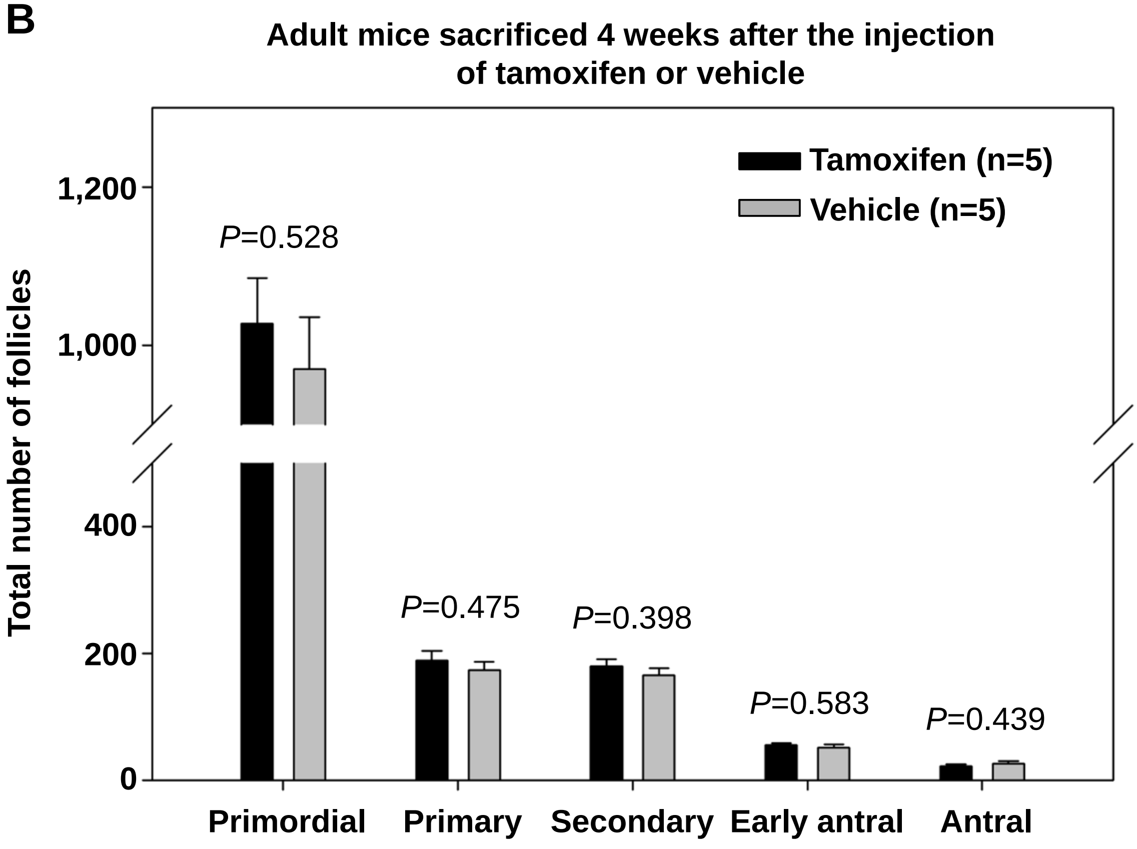
Adult mice sacrificed 4 weeks after the injection
of tamoxifen or vehicle
Tamoxifen (n=5)
Vehicle (n=5)
Total number of follicles
1,200
1,000
400
200
0
P=0.528
P=0.475
P=0.398
P=0.583
P=0.439
Primordial
Primary
Secondary
Early antral
Antral

Fig. S6. Follicular development was not altered by the dosage of tamoxifen injected in adulthood.

Three-month-old *R26R* females were given daily injections of tamoxifen (80 mg/kg BW) or vehicle for 3 consecutive days. At 2 and 4 weeks after the injection, ovarian follicles at different stages of development, including primordial, primary, secondary, early antral, and antral follicles, were quantified in all sections per ovary based on the well-accepted standards.

(**A**) At 2 weeks after the injection, no significant differences in the numbers of follicles at any developmental stage were seen between tamoxifen- and vehicle-injected females.

(**B**) At 4 weeks after the injection, no significant differences in the numbers of follicles at any developmental stage were seen between tamoxifen- and vehicle-injected females. Values are means ± S.E.M. The *P* values are shown above the bars.
