# Supplementary material for: Two classes of ovarian primordial follicles exhibit distinct developmental dynamics and physiological functions
Source: Hum Mol Genet. 2013 Oct 1;23(4):920–8. doi: 10.1093/hmg/ddt486 (PMC3900105; doi:10.1093/hmg/ddt486)
Supplement: Supplementary Data [file supp_ddt486_ddt486supp.docx]

**Supplementary Text**

**Evaluation of the influence of tamoxifen on the development of ovarian follicles**

To evaluate the potential effect of tamoxifen administration at the embryonic stage, we injected tamoxifen at 15 mg/kg BW or vehicle alone into pregnant *mT/mG* females at E16.5. Because the primordial follicle pool is established by PD8 in mouse ovaries (S1), we first analyzed the initial primordial follicle pool in pups at PD8. As shown in Fig. S3A, quantification of ovarian follicles showed that the numbers of primordial follicles in pups from tamoxifen- or vehicle-injected mothers were similar. The numbers of growing follicles (primary and secondary stages) were also comparable between the two groups. In addition, no abnormalities in ovarian morphology were observed in pups born from tamoxifen-injected mothers at either PD8 (Fig. S3B) or 2 months of age (Fig. S3C).

Thus, injection of 15 mg/kg BW tamoxifen to pregnant females at E16.5 did not alter the formation or development of ovarian follicles in their pups.

To evaluate the potential influence of tamoxifen on ovarian development during adulthood, we injected tamoxifen (80 mg/kg BW daily for 3 consecutive days) or vehicle alone into 3-month-old female *R26R* mice. We first quantified follicles at various developmental stages 2 weeks after tamoxifen injection. As shown in Fig. S6A, no significant difference (*P* > 0.05) was observed in the numbers of primordial or primary follicles between tamoxifen- and vehicle-injected females, indicating that the survival and activation of primordial follicles were not affected by the amount of tamoxifen injected. The numbers of follicles from secondary to antral stage were also comparable (*P* > 0.05) between the two groups, indicating that the tamoxifen dosage used did not alter the further development of the follicles. Similar numbers of follicles at various developmental stages were also observed in tamoxifen- and vehicle-injected groups at 4 weeks after tamoxifen injection (Fig. S6B).

Therefore, in accordance with previous reports (S2, S3), the development of ovarian follicles in adult female mice was not affected by administration of tamoxifen at a daily dose of 80 mg/kg BW for 3 consecutive days.

**Supplementary References:**

S1. Pepling, M.E. (2012) Follicular assembly: mechanisms of action. *Reproduction,* **143**, 139-149.

S2. John, G.B., Gallardo, T.D., Shirley, L.J. and Castrillon, D.H. (2008) Foxo3 is a PI3K-dependent molecular switch controlling the initiation of oocyte growth. *Dev. Biol*., **321**, 197-204.

S3. Hayashi, S. and McMahon, A.P. (2002) Efficient recombination in diverse tissues by a tamoxifen-inducible form of Cre: a tool for temporally regulated gene activation/inactivation in the mouse. *Dev. Biol*., **244**, 305-318.
